# Modelling Microstructural Deformation and the Failure Process of Plastic Bonded Explosives Using the Cohesive Zone Model

**DOI:** 10.3390/ma12223661

**Published:** 2019-11-07

**Authors:** Kaida Dai, Baodi Lu, Pengwan Chen, Jingjing Chen

**Affiliations:** 1 State Key Laboratory of Explosion Science and Technology, Beijing Institute of Technology, Beijing 100081, China; 3120185134@bit.edu.cn (B.L.); pwchen@bit.edu.cn (P.C.); 2 Shanghai Space Propulsion Technology Research Institute, Shanghai Academy of Spaceflight Technology, China Aerospace Science and Technology Corporation, Shanghai 200125, China; pingjing10@163.com

**Keywords:** polymer-bonded explosives, mechanical behavior, cohesive zone model, bilinear softening law, failure mechanism

## Abstract

A microstructure finite element method combining the cohesive zone model (CZM) is used to simulate the mechanical behavior, deformation, and failure of polymer-bonded explosive (PBX) 9501 under quasi-static loading. PBX 9501 consists of Cyclotetramethylene tetranitramine (HMX) filler particles with a random distribution packaged in a polymeric binder. The particle is treated as elastic and the binder as viscoelastic. Cohesive elements with a bilinear softening law are inserted into the particle/binder interface, the HMX particle, and the binder to study the interface’s debonding and failure evolution. Macroscopic stress–strain curves homogenized across the microstructure under tension and compression with different strain rates are basically consistent with the experimental data. The interface debonding approximately vertical to the loading direction is the primary failure mechanism under tension, while shear failure along the interfaces and particle fracture plays a significant role under compression. The effects of interface strengths and strain rates on the performance of PBX 9501 are also evaluated. The tensile and compressive strengths are dependent on the interface strength and strain rate, but the failure paths are insensitive. This model is shown to accurately predict macroscopic responses and improve our understanding of the relationship between the mechanical behavior and microstructure of PBX 9501.

## 1. Introduction

Polymer-bonded explosives (PBXs) are a type of highly filled particulate composite, generally composed of high-energy single-compound explosive particles and a polymeric binder [1,2]. PBXs have been widely used in military and civil products, such as missiles, rockets, and anti-tank bombs. The little insert polymer binder is used to reduce sensitivity and to make the material safer to handle. However, the binder can strongly affect the mechanical and fracture properties of PBXs. Debonding of the particle/binder interface, and even failure of the microstructure can arise as PBXs may be subjected to tension and compression loading. The failure caused by different stimuli influences the mechanical properties and even detonation behavior of PBXs. Understanding the microstructural mechanical properties and failure mechanisms of PBXs are important for their design and safe application.

Many experimental studies of the micromechanical properties and failure mechanisms of PBXs have been conducted in recent years. The Brazilian test with real-time microscopic observations was utilized to investigate the material’s microstructural deformation evolution and failure mechanisms under quasi-static tension [3,4,5,6]. Different failure modes, including interface debonding, binder rupture, and (rarely) crystal fracture have been reported. Palmer et al. also analyzed the critical stress of debonding for PBXs. Skidmore et al. and Chen et al. used uniaxial compression experiments to study the evolution of the microstructures and failure mechanisms of PBXs under quasi-static compression and found that crystal fracture plays an important role in failure [7,8]. At present, some optical techniques have been used to quantitatively measure the deformation of the microstructures of PBXs and also study their mechanical properties. Rae et al. and Goldrein et al. measured the deformation fields and strain fields of the disc central micro-region using moiré interferometry and further revealed the material’s deformation and damage processes [9,10]. Williamson et al. and Zhou et al. utilized the Digital Image Correlation (DIC) method to discover that interface debonding dominates the failure of PBXs in Brazilian tests [11,12,13]. The damage evolution mechanisms of PBX simulants under a creep test and uniaxial compression were also found by DIC observations [14,15].

It is very important to predict the debonding between particles and adhesives in explosives because more complex stress distribution is related to debonding failure. The interface failure is well characterized by the cohesive zone model (CZM), in which a traction-displacement constitutive law can reflect the discontinuities in the materials [16,17]. Recently, many researchers have paid much attention to the bilinear cohesive law proposed by Geubelle and Baylor [18]. A CZ model was used by Zhong et al. to study the debonding between the particle and matrix, as well as the effect of particle size on the damage evolution in a particle-filled elastomer [19]. They reported that initial debonding tends to start from the edges of larger particles. Arora et al. used a finite element method to investigate the damage process of PBX-1 under tension [20]. A set of finite element models, including 2D polygons of actual SEM images, idealized 2D circles, and 3D spheres of particles, were used in the analysis. The results from these studies show that the mechanical properties are highly sensitive to cohesive law parameters but are insensitive to the model size. Seidel et al. used a linear viscoelastic cohesive zone model for the binder and Voronoi tessellation for the explosive crystals to simulate the mechanical response of LX17 [21]. Barua et al. embedded cohesive elements throughout the microstructure to quantify the thermomechanical response, as well as failure, of PBXs at high strain rates [22,23]. They highlighted that the threshold of fracture initiation decreased with an increase in the volume fractions of HMX granules. Bimodal distributions of granule sizes improved mechanical integrity better than monomodal distributions. Wu et al. employed a rate-dependent viscoelastic cohesive law for the particle/binder interface to investigate the effects of interface debonding on the fracture of PBX 9501 and validated their simulated results with experimental data [24]. Wang et al. further developed damage models for HMX crystals, as well as their binders and their interfaces, to analyze the dynamic mechanical behavior and damage evolution of PBX 9501 at a mesoscale level [25]. At present, the application of the CZ model to the study of the deformation and failure of PBXs under quasi-static compression loading is rarely found in the literature.

The characteristics of PBX 9501 are complex, with a higher than usual particle volume fraction. The actual PBX microstructure and the idealized microstructure of randomly distributed circular or polygonal particles have been reported in the literature; the range of the particle’s volume fraction is from 0.42 to 0.74. In the present study, the Voronoi tessellated method is used to generate a microstructure with randomly distributed polygonal particles and a very high-volume fraction. A bilinear cohesive law with normal (mode I) and tangential direction (mode II) is developed, and cohesive elements are inserted into the microstructure along the element boundaries of the particle, binder, and particle/binder interface to study the interface’s debonding, failure evolution, and shear fracture. Both tension and compression simulations are carried out for the different mechanical responses and failure mechanisms under different stress states. The effect of the strain rate and interface strength are also considered. The objective is to greatly improve the understanding of debonding, damage, and failure mechanisms under quasi-static loading at the micro level.

## 2. Material Constitutive Models

### 2.1. HMX Particles

It is generally believed that HMX is brittle at ambient pressure, so it undergoes very little plastic deformation [26]. Hence, we made the simplifying assumption that HMX can be regarded as a linearly isotropic elastic material with Young’s modulus of 13.375 Mpa and a Poisson’s ratio of 0.32 in this study.

### 2.2. Binder Material

Estane 5703, considered as a polymer binder, is used in PBX 9501. A series of experiments were carried out on Estane 5703 to measure its mechanical properties at strain rates of 10^−4^ s^−1^ to 10^3^ s^−1^ [27]. The material shows viscoelastic properties with a dependence on the strain rate. A 22 element Prony series is used to describe the variation of the shear modulus *G* with the relaxation time. The Prony series formulation is

(1) $$G(\tau )=G_{\infty }+\sum_{i=1}^{N_{p}}G_{i}e^{-\frac{\tau }{\tau_{i}^{P}}}=G_{0}(g_{\infty }+\sum_{i=1}^{N_{p}}g_{i}e^{-\frac{\tau }{\tau_{i}^{P}}})$$

(2) $$G_{0}=G_{\infty }+\sum_{i=1}^{N_{p}}G_{i}$$

where $G_{0}$ is the instantaneous shear modulus at reference temperature $T_{0}$, $G_{\infty }$ is the steady-state shear modulus, $g_{i}=G_{i}/G_{0}$ is the relative modulus of the ith term, $N_{p}$ is the number of terms in the Prony series, and $\tau_{i}^{p}$ is the relaxation times. The terms for the Prony series defining G(t) are listed in Table 1.

The stress–strain relationship of the generalized viscoelastic body can be expressed by an integral form

(3) $$\sigma (t)=\int_{0}^{t}2G(\tau -\tau^{'})\frac{\partial \varepsilon^{D}}{\partial \tau^{'}}d\tau^{'}+\int_{0}^{t}K(\tau -\tau^{'})\frac{\partial \varepsilon^{H}}{\partial \tau^{'}}d\tau^{'}$$

where σ is the Cauchy stress, $\varepsilon^{D}$ and $\varepsilon^{H}$ are the deviatoric and hydrostatic portions of the Eulerian strain tensor, and t and τ are the physical and reduced times, respectively. The bulk modulus K is a constant, as in [24,27].

### 2.3. CZM with a Bilinear Constitutive Law

**Figure 1:**
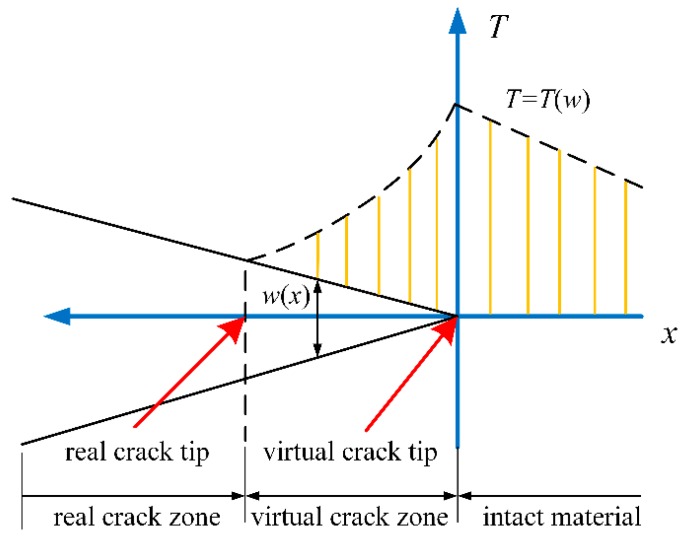
Cohesive zone model.

**Figure 2:**
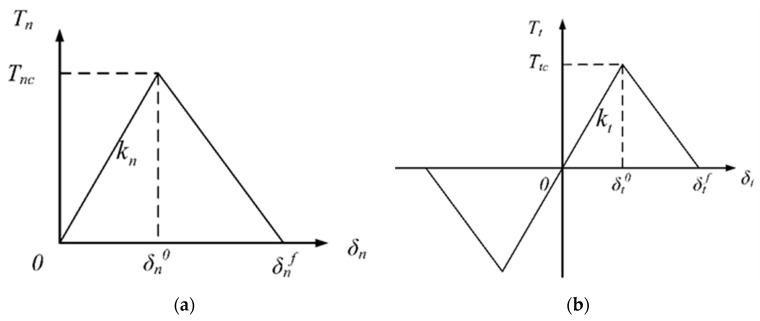
Bilinear traction–separation law for the cohesive zone model; (**a**) normal direction (mode I) and (**b**) tangential direction (mode II).

A cohesive zone model, assuming an imaginary zone in front of the real crack tip, is used to predict the crack in the PBX samples. As shown in Figure 1**,** the tip of this zone is the boundary point between the intact material and the damaged material. In this zone, the traction linearly increases from zero at the rear crack tip to its maximum at the virtual crack tip. A typical bilinear traction-separation law (as illustrated in Figure 2) is used to characterize the nonlinear relationship between the traction and separation in the cohesive model. Figure 2a shows the curve of the traction’s normal component $T_{n}$ with the crack’s opening displacement, while Figure 2b shows the curve of the traction’s tangential component $T_{t}$ with the crack’s sliding displacement. The traction first increases linearly, with separation displacement on the undamaged cohesive crack’s surface, and then decreases monotonically after damage and finally reduces to zero after the entire separation.

The $T_{n}$ and $T_{t}$ can be expressed as

When $\delta_{n}>0$,

(4) $$T_{n}=\left\{ \begin{array}{ll} \frac{T_{nc}}{\delta_{n}^{0}}\delta_{n}, & \left(\delta_{n}\leq \delta_{n}^{0}\right)\\ \frac{T_{nc}}{\delta_{n}^{f}-\delta_{n}^{0}}(\delta_{n}^{f}-\delta_{n}) & \left(\delta_{n}^{0}<\delta_{n}\leq \delta_{n}^{f}\right) \end{array}\right.$$

(5) $$T_{t}=\left\{ \begin{array}{ll} \frac{T_{tc}}{\delta_{t}^{0}}\delta_{t}, & \left(\delta_{t}\leq \delta_{t}^{0}\right)\\ \frac{T_{tc}}{\delta_{t}^{f}-\delta_{t}^{0}}(\delta_{t}^{f}-\delta_{t}) & \left(\delta_{t}^{0}<\delta_{t}\leq \delta_{t}^{f}\right) \end{array}\right..$$

When $\delta_{n}=0$,

(6) $$T_{t}=\left\{ \begin{array}{ll} \frac{T_{tc}}{\delta_{t}^{0}}\delta_{t}, & \left(\delta_{t}\leq \delta_{t}^{0}\right)\\ \frac{T_{tc}}{\delta_{t}^{f}-\delta_{t}^{0}}(\delta_{t}^{f}-\delta_{t}) & \left(\delta_{t}^{0}<\delta_{t}\leq \delta_{t}^{f}\right) \end{array}\right.$$

where n and t are unit vectors along the normal and tangential directions of the cohesive crack surface, and δ is the cohesive crack surface separation displacement vector. $\delta_{n}=n\cdot \delta$ and $\delta_{t}=t\cdot \delta$ are the normal and tangential separation displacement components. $T_{nc}$ and $T_{tc}$, respectively, represent the maximum normal and tangential cohesive strength. $\delta_{n}^{0}$ and $\delta_{t}^{0}$, respectively, represent the normal and tangential separation displacement corresponding to $T_{nc}$ and $T_{tc}$. $\delta_{n}^{f}$ and $\delta_{t}^{f}$ are the normal and tangential critical relative separation displacement, respectively. $k_{n}=\frac{T_{nc}}{\delta_{n}^{0}}$ and $k_{t}=\frac{T_{tc}}{\delta_{t}^{0}}$ are the normal and tangential initial stiffness coefficient, respectively.

The cohesive zone model involves three parameters: the cohesive strength T, the separation displacement δ, and the initial stiffness coefficient k. The separation displacement δ is also determined by the cohesive fracture energy G, which is equal to the area under the traction–separation curves (see Figure 2). $G_{nc}=\int_{0}^{\delta_{n}^{f}}T_{nc}d\delta_{n}=\frac{T_{nc}\delta_{n}^{f}}{2}$ and $G_{tc}=\int_{0}^{\delta_{t}^{f}}T_{tc}d\delta_{t}=\frac{T_{tc}\delta_{t}^{f}}{2}$ represent the normal and tangential fracture energy.

The damage, activated in terms of a maximum stress criterion, can be expressed as

(7) $$\max\left\{\frac{\langle T_{n}\rangle }{T_{nc}},\frac{\langle T_{t}\rangle }{T_{tc}}\right\}=1$$

where 〈〉 is the Macaulay bracket, defined as

(8) $$\langle T_{n}\rangle =\{\begin{array}{cc} T_{n} & T_{n}\geq 0\;for\;tension\\ 0 & T_{n}<0\;for\;compression \end{array}.$$

**Figure 3:**
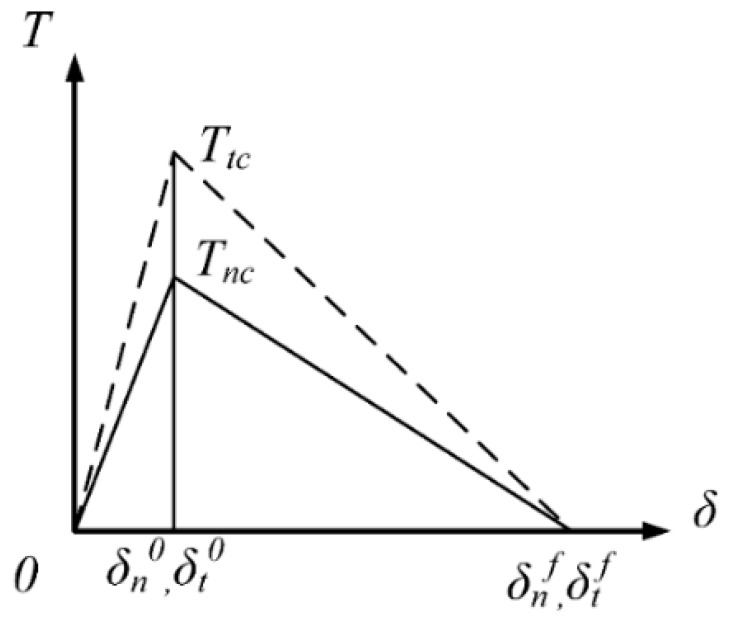
An assumption so-called “equal ratio attenuation”.

The cohesive element is represented by the inserted particles, binders, and particle/binder interface along the element boundaries. The normal and tangential parameters need to be determined for the three types of cohesive elements. The calculation of critical separation displacement caused by different normal and tangential initial stiffness is very complicated. Li et al. proposed an assumption so-called “equal ratio attenuation” to simplify the constitutive model and facilitate the determination of the parameters [28,29]. As shown in Figure 3, as the tangential and the normal displacement are the same, both stiffnesses always maintain the same ratio, therefore the same value of separation displacement is assumed for both the normal direction (mode I) and the tangential direction (mode II).

**Figure 4:**
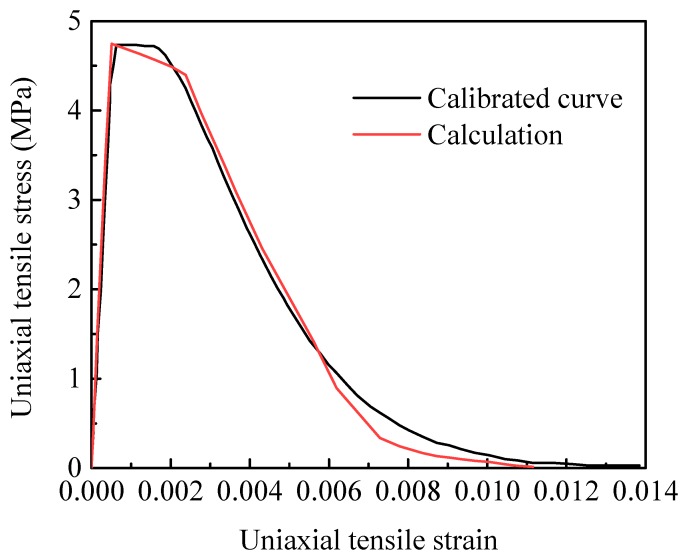
Calculated and measured stress-strain curves for HMX under uniaxial tension loading at strain rate of 10^−3^ s^−1^. Calibrated curve is due to Wu et al. [24].

According to Wu [24], the tensile strength of 4.754 MPa is taken as the maximum normal cohesive strength $T_{nc}$ of the cohesive elements in the HMX particles, while the corresponding separation displacement $\delta_{n}^{0}$ is 2.38 μm, and the normal initial stiffness of $k_{n}=2\mathrm{GPa}/\mu m$. The compression strength $\sigma_{c}$ of 260 MPa is given for HMX in the literature. The maximum tangential cohesive strength, considered as the shear strength, is 131 MPa, which can be calculated as $T_{tc}=\frac{\sigma_{c}+0.3T_{nc}}{2}$, and then the tangential initial stiffness is $k_{t}=55\mathrm{GPa}/\mu m$. The same critical separation displacement proposed in the literature is used for both the normal direction (mode I) and tangential direction (mode II) (i.e., $\delta_{n}^{f}=\delta_{t}^{f}=5\mu m$). Figure 4 shows the calculated and measured stress-strain curves for HMX crystal at uniaxial tensile strain rate of 10^−3^ s^−1^. It is possible to accurately characterize the HMX crystal behavior with the parameters from literature.

**Figure 5:**
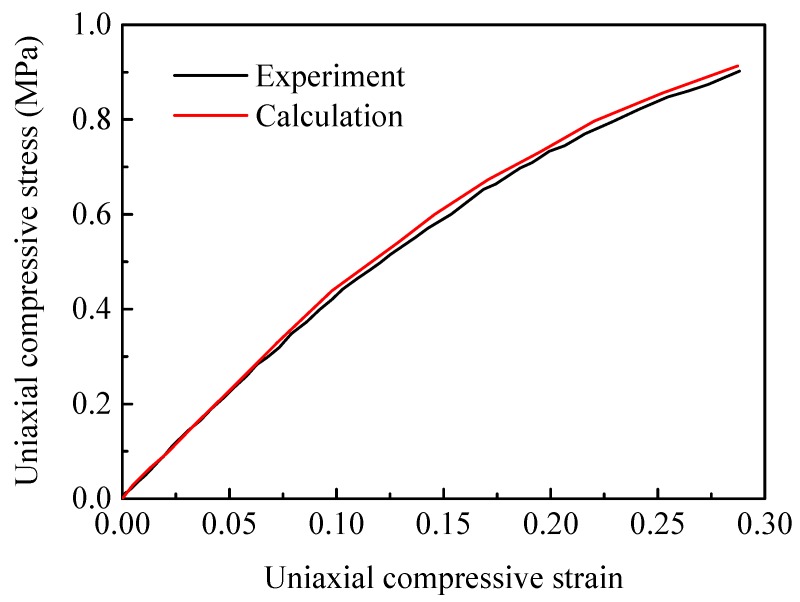
Calculated and measured stress-strain curves of Estane 5703 at compressive strain rate of 10^−3^ s^−1^. Experiment by Cady et al. [30].

The literature values for the cohesive elements in the binder are used [22,23]. A cohesive strength of 5 MPa and an initial stiffness of 1 GPa/μm are given for the normal direction (mode I), while a cohesive strength of 38.4 MPa and initial stiffness of 7.68 GPa/μm are given for the tangential direction (mode II). The normal and tangential critical separation displacements have the same values of $\delta_{n}^{f}=\delta_{t}^{f}=5\mathrm{mm}$. Figure 5 presents the calculated and measured compressive stress-strain curves of the binder material at strain rate of 10^−3^ s^−1^. From the figure, the simulation result is slightly higher than the experimental value. More experimental data of the Estane 5703 under quasi-static loading at the different strain rates are needed to validate the rate sensitivity.

**Figure 6:**
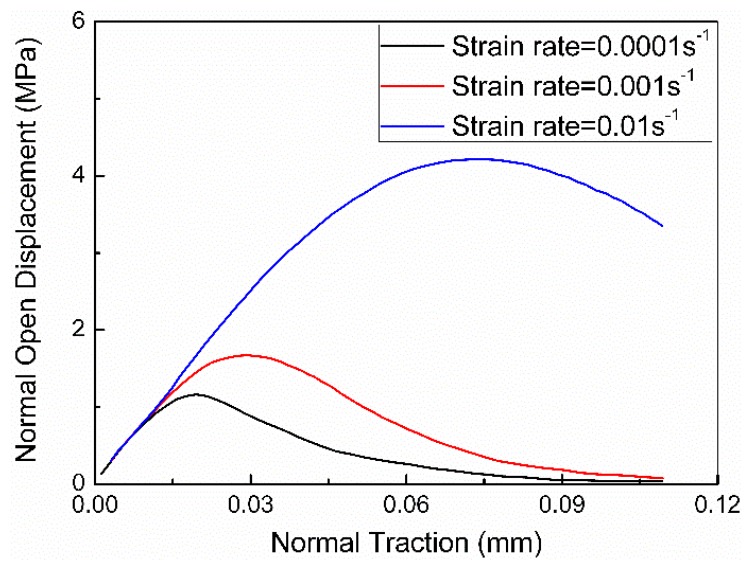
Normal traction–separation curves at different loading rates.

The values for the cohesive elements in the particle-binder interface are taken from the work of Tan et al. [31], who used an experimental method to study a cohesive law for the particle/binder interface in PBX 9501. The normal cohesive strength is $T_{nc}=1.66\;\mathrm{MPa}$ and the normal initial stiffness is $k_{n}=1.55\;\mathrm{GPa}/\mu m$. The normal and tangential separation displacements reach the critical value of $\delta_{n}^{f}=\delta_{t}^{f}=0.11\;\mathrm{mm}$. Dienes and Kershneer measured the energy consumption per unit area to be about 195 J/m^2^ during the binder tearing process [32]. The experimental results show that the mode II crack on the interface is always accompanied by a tearing of the binder. Herein, we take the tangential cohesive fracture energy $G_{tc}=195\;{J/m}^{2}$, so the calculated value of $T_{tc}=3.55\;\mathrm{MPa}$, and the tangential initial stiffness of $k_{t}$ is taken as 3.3 GPa/μm. Figure 6 shows the normal traction–separation curves of the particle/binder interface at different loading rates [24]. The cohesive strength and fracture energy increased with an increase in the loading rate. Therefore, we should consider incorporating the rate-dependence of the interface into the model. Table 2 summarizes the parameters used for the three types of cohesive elements. Further calibration for the CZ parameters will be performed using experimental data for PBX 9501 in Section 3.3.

As mentioned in the literature, initially inserted bilinear CZ models reduce cohesive-surface-induced stiffness when a finite initial stiffness is used in the cohesive law [33,34,35]. A sufficiently large initial stiffness (*k =* 1–55 GPa/μm) and a finite element size of 8 μm are used to solve this problem in our work.

## 3. Computational Model

### 3.1. Microstructure Model of PBX

**Figure 7:**
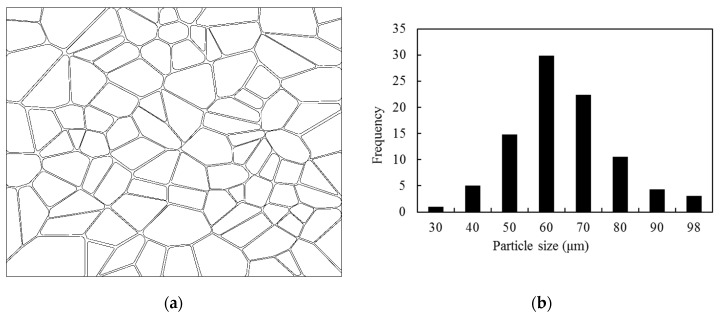
Microstructure of a polymer-bonded explosive (PBX), (**a**) a Voronoi tessellation model, and (**b**) the particle size distribution.

The PBX 9501 microstructure selected here consists of an HMX particle (approximately 95% by mass) and a typical binder material—Estane 5703. Voronoi tessellation is applied to generate random distributions for the HMX particle polygons of various shapes and sizes, as shown in Figure 7. The PBX microstructure is a 1 mm × 0.8 mm 2D model consisting of 98 HMX particles with a size distribution from 25 μm to 98 μm and an Estane 5703 binder with a width of 8 μm. The densities of HMX and Estane 5703 are 1.58 g/cm^3^ and 0.9 g/cm^3^, respectively. The model contains 91% HMX by volume fraction, which is equivalent to a 94.7% mass fraction. On account of the simulation results of the model with 98 and 200 particles, we verified that 98 particles can provide reasonable simulation results, so we did not consider using more particles.

**Figure 8:**
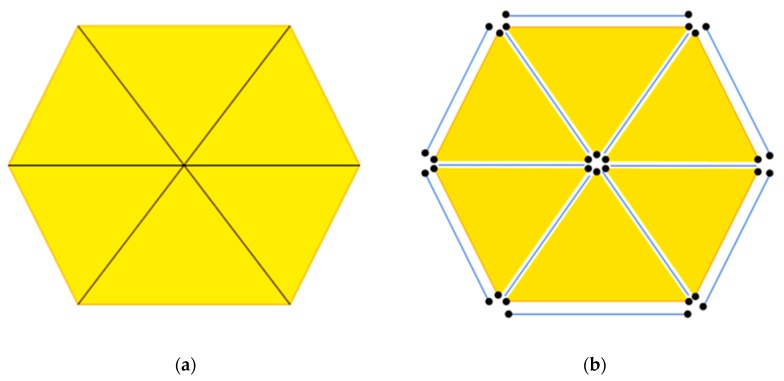
The cohesive element inserting algorithm; (**a**) six triangular elements before inserting and (**b**) reset nodes and cohesive elements after inserting.

**Figure 9:**
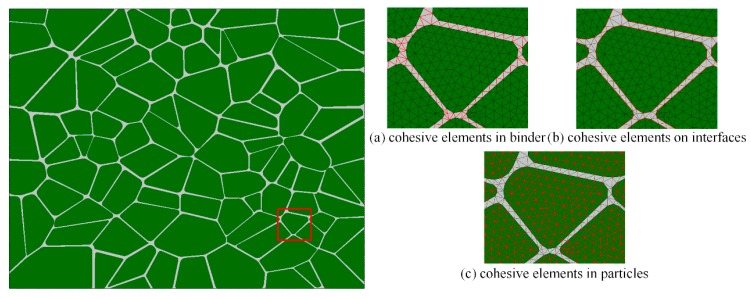
Cohesive elements in the finite element model.

The obtained microstructure model is introduced into ABAQUS/Explicit finite element code (ABAQUS, 2016) to partition the CPS3 elements (the triangular plane stress elements) with a mesh size of 8 μm [36]. As mentioned in the literature [17,22,35], cohesive elements are embedded along all finite element boundaries as part of the physical model to predict fracture patterns and the fracture outcome in our analysis. We referred to the insertion procedure proposed by Yang et al. By using our program, COH2D4 elements (a 4-node cohesive element with zero in-plane thickness) are embedded into the model along the CPS3 element boundaries [37]. A node shared by six triangular elements is used as an example to illustrate the embedding process, as shown in Figure 8a. This node is first copied and reset by six separate nodes at the same location (see Figure 8b), and then the reset nodes are connected along the triangular element sides to generate six zero-thickness cohesive elements. This model required a total of 118,377 elements. The three types of cohesive elements are highlighted in red, as shown in Figure 9.

### 3.2. Loading and Boundary Conditions

**Figure 10:**
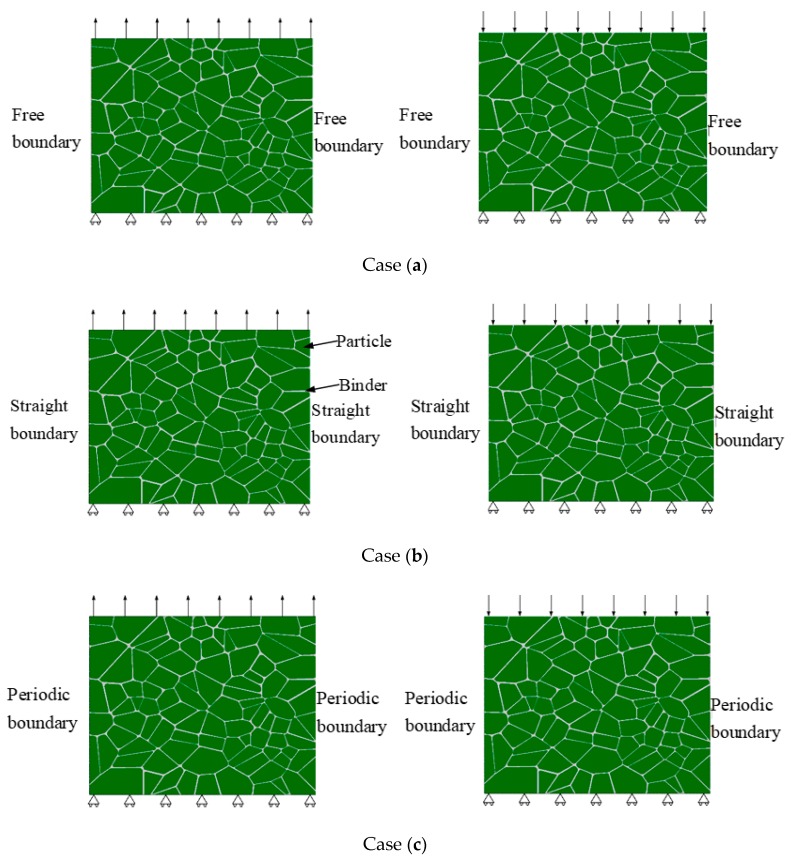
Loading and the three boundary conditions; case (**a**) traction free vertical edges, case (**b**) straight vertical edges, and case (**c**) periodic vertical edges.

The macroscopic response is strongly affected by the boundary conditions. Three different cases, including (a) traction free vertical edges, (b) straight vertical edges, $u\left(0,y\right)=u\left(0,L_{y}\right),u\left(L_{x},y\right)=u\left(L_{x},L_{y}\right)$, and (c) periodic vertical edges $u\left(L_{x},y\right)=u\left(0,y\right)+u\left(L_{x},0\right)$, where u is displacement and L is length of model, were investigated to simulate the tensile and compressive loading of the model. The loading and boundary conditions are shown in Figure 10. The vertical displacement loading control is applied at the top surface to simulate a uniaxial tension and compression condition. The bottom surface is constrained in the vertical direction.

**Figure 11:**
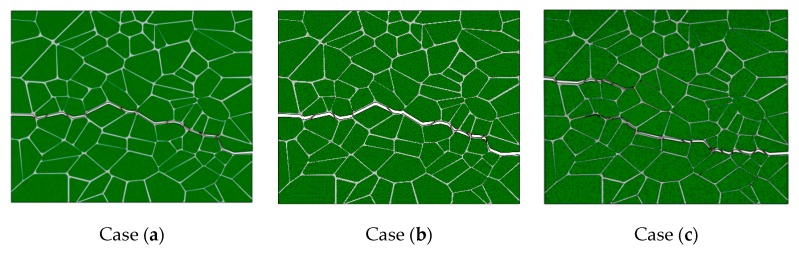
Simulation results for the three boundary conditions: (**a**) traction free vertical edges; (**b**) straight vertical edges; (**c**) periodic vertical edges; under tensile loading.

**Figure 12:**
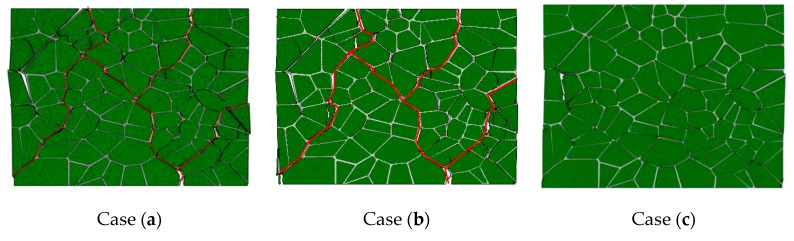
Simulation results for the three boundary conditions: (**a**) traction free vertical edges; (**b**) straight vertical edges; (**c**) periodic vertical edges; under compression loading.

The results presented in Figure 11 and Figure 12 show that the choice between cases (a) and (b) of boundary conditions does not affect the outcome of a given simulation. Case (c) shows some difference due to periodic boundary conditions are generally applied to repeatable units of materials, and the geometry in our study although representative of the material, is random and not repeatable. The same calculated results have been also proposed by Arora et al. [20]. Therefore, all further simulations are performed using case (b) boundary condition.

The average stress ${\bar{\sigma }}_{y}$ is the average value of the stress of all the elements except the cohesive elements in the loading direction, ${\bar{\sigma }}_{y}=(\sum_{i}\sigma_{yi}S_{i})/S$. The strain is calculated by the displacement $u_{y}$ on the top surface, $\varepsilon_{y}=u_{y}/L_{y}$, $L_{y}$ is the length of model at Y direction.

### 3.3. Model Calibration

**Figure 13:**
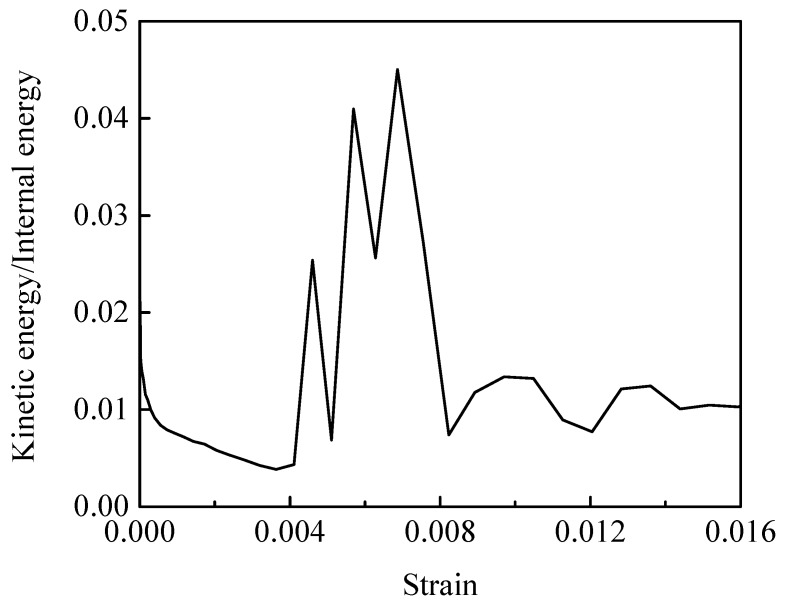
The ratio of kinetic energy to internal energy.

The simulations were performed using ABAQUS/Explicit. A massing scale ensured that the kinetic energy was negligible compared to the internal energy ensuring the simulation was quasi-static. As shown in Figure 13, the ratio of kinetic energy to internal energy is less than 5% during the loading process. The effect of element size is analyzed by three different element sizes with 5 μm, 8 μm and 10 μm. The calculated peak stress and failure strain at a tensile strain rate of 10^−3^ s^−1^ and compressive strain rate of 10^−2^ s^−1^ are listed in Table 3. The relative error in the peak stress and failure strain is less than 5.13% of the value for 5 μm element size. Considering the balance between efficiency and accuracy, the element size of 8 μm is chosen for further simulations.

**Figure 14:**
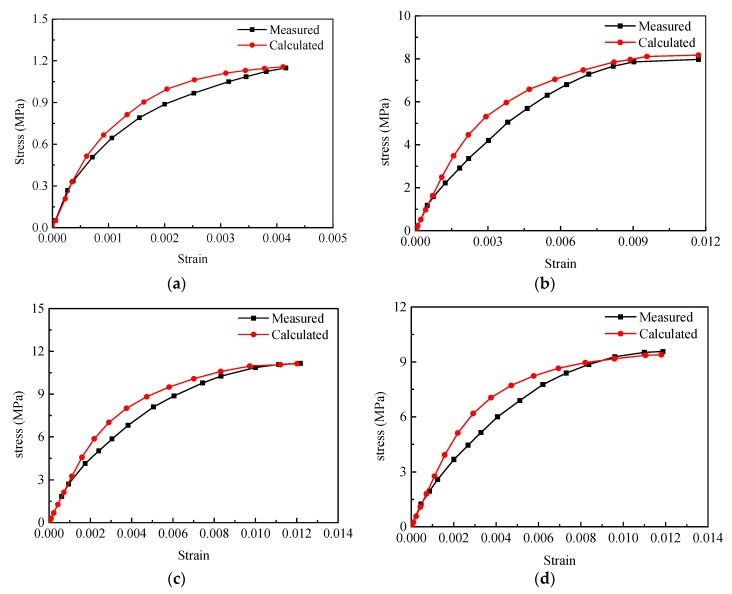
Calculated and measured stress–strain curves for PBX 9501. (**a**) tension; (**b**) compressive strain rate of 10^−4^ s^−1^; (**c**) compressive strain rate of 10^−3^ s^−1^; (**d**) compressive strain rate of 10^−2^ s^−1^.

The measured tensile and compressive stress–strain curves of PBX 9501 from Wu et al. and Rangaswamy et al. have been used to calibrate the simulation results at different strain rates [24,38]. Since the data obtained from the literature only include the stress–strain curves before the damage of PBX 9501, we compared the curves of the strain from 0 to the failure strain. Figure 14 shows a comparison of the measured and simulated stress–strain curves of PBX 9501 at three strain rates of 10^−4^ s^−1^, 10^−3^ s^−1^, and 10^−2^ s^−1^. The measured and calculated responses under uniaxial tension (in Figure 14a) and compression (Figure 14b–d)) are in good agreement with each other. The tensile strength, failure strain, and elastic modulus calculated at a strain rate of 10^−3^ s^−1^ are about 1.16 MPa, 0.0041, and 0.97 GPa, which are close to the values of 1.15 MPa, 0.0042, and 1 GPa obtained from [24]. At the three strain rates of 10^−4^ s^−1^, 10^−3^ s^−1^, and 10^−2^ s^−1^, the compressive strengths obtained by the numerical simulation are 8.16 MPa, 9.36 MPa, and 11.13 MPa, respectively, which are close to the experimentally obtained compressive strengths of 7.97 MPa, 9.57 MPa, and 11.15 MPa. The failure strains are 0.0117, 0.0118, and 0.012, which are close to the experimentally obtained failure strains of 0.0117, 0.0119, and 0.0122.

## 4. Results and Discussion

### 4.1. Simulations for Uniaxial Tension

#### 4.1.1. Stress–Strain Response and Failure Mechanism

**Figure 15:**
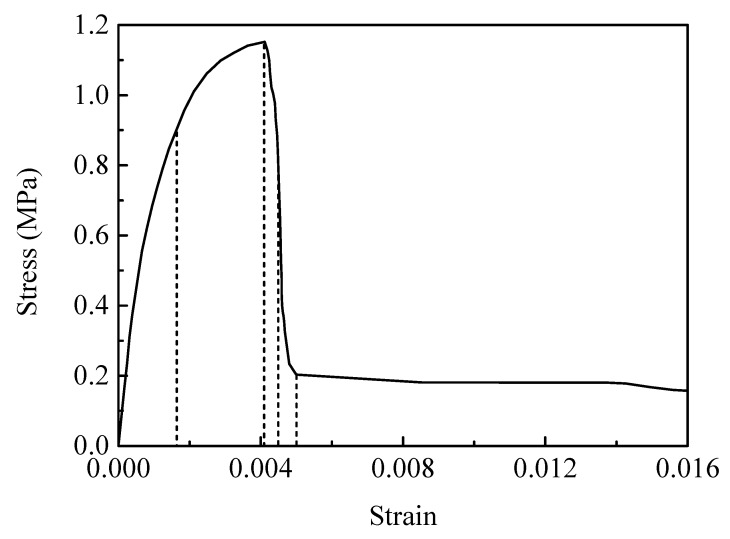
The calculated tensile stress–strain curve of PBX 9501 at a strain rate of 10^−3^ s^−1^_._

Figure 15 shows the macroscopic stress–strain curve calculated at a strain rate of 10^−3^ s^−1^. The stress increases monotonously and nonlinearly up to the peak stress, and then sharply drops due to the failure from the interface debonding. We noted that the failure strain in uniaxial quasi-static tension is only about 0.0041, revealing the brittle, fractural nature of PBX 9501.

**Figure 16:**
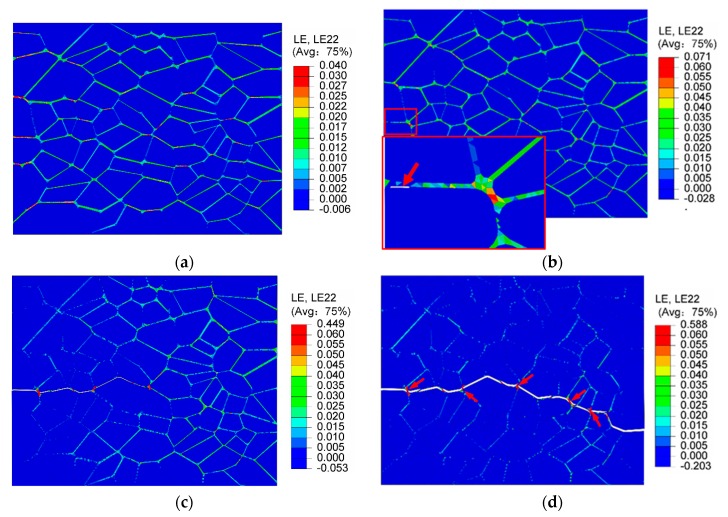
The true strain contours of the y-direction $\varepsilon_{y}$ and failure evolution at macroscopic strains: (**a**) 0.16%, (**b**) 0.41%, (**c**) 0.45%, and (**d**) 0.5%.

The true strain contours of y-direction $\varepsilon_{y}$ and the failure evolution at the macroscopic strains of 0.16%, 0.41%, 0.45%, and 0.5% (marked by dash line in Figure 15) are shown in Figure 16. The soft binder accommodates a great amount of the deformation throughout the process. The strain distribution is heterogeneous and results from the microstructure’s features (i.e., the random particle size shapes and interface debonding behavior). Localized strain concentrations labeled in red can be found in some regions around the particle sides (Figure 16a), yet no microcracks are formed at a macroscopic strain of 0.16%. At a macroscopic strain of 0.41% (shown in Figure 16b), a microcrack, as indicated by the arrows, is observed along the interface with a weaker strength as a result of interface debonding, thereby lowering the material’s load-carry capacity and contributing to material softening. The form of the microcrack reduces the effective mechanical properties of the microstructure [39]. With the increasing of loading, greater interface debonding makes the microcrack grow continuously (indicated by Figure 16c). It can be seen that the strain inside the binder on both sides of the crack reduces and redistributes itself. At a macroscopic strain of 0.5%, a single main macrocrack is formed roughly vertical to the loading direction (Figure 16d). However, it is interesting to note that the local maximum strain inside the binder on the crack path reaches 0.588. Residual binders, as marked by the arrows, allow the structure to continue to withstand certain stresses. This also explains why the macrostress of the stress–strain curve in Figure 9 is maintained at about 0.1 MPa. From Figure 16, we can see that the failure mechanism of PBX 9501 in uniaxial quasi-static tension is predominantly interface debonding, which is similar to the phenomenon monitored in the real microscopic examination by Rae et al. and Chen et al. [5,8].

#### 4.1.2. Effect of the Interface Strength

**Figure 17:**
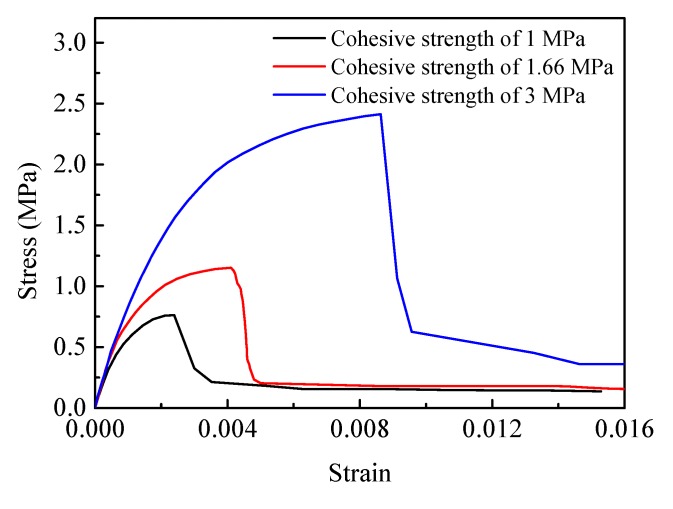
Tensile stress–strain curves of PBX 9501 with different interface strengths at a strain rate of 10^−3^ s^−1^.

**Figure 18:**
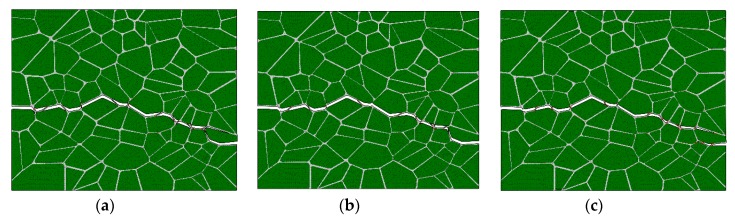
Crack path of PBX 9501 under uniaxial tension at a strain rate of 10^−3^ s^−1^ for different interface cohesive strengths: (**a**) 1 MPa, (**b**) 1.66 MPa, and (**c**) 3 MPa.

The interface cohesive strength affecting the mechanical properties of PBXs has been reported in some of the literature. Three interface cohesive strengths of 1, 1.66, and 3 MPa are used for the model illustrated in Figure 10b to investigate the mechanical behavior of PBX 9501 under uniaxial tension. The calculated stress–strain curves for the different interface cohesive strength models are shown in Figure 17. As the interface cohesive strength increases from 1 to 3 MPa, the tensile strength increases from 0.78 to 2.41 MPa, and the failure strain increases from 0.0035 to 0.0086. It is remarkable that the predicted crack path is almost identical, as shown in Figure 18a–c. This result suggests that PBX 9501’s tensile mechanical properties always are related to its interface cohesive strength, while the failure path is independent.

### 4.2. Simulations of Uniaxial Compression

#### 4.2.1. Mechanical Response and Failure Mechanism

**Figure 19:**
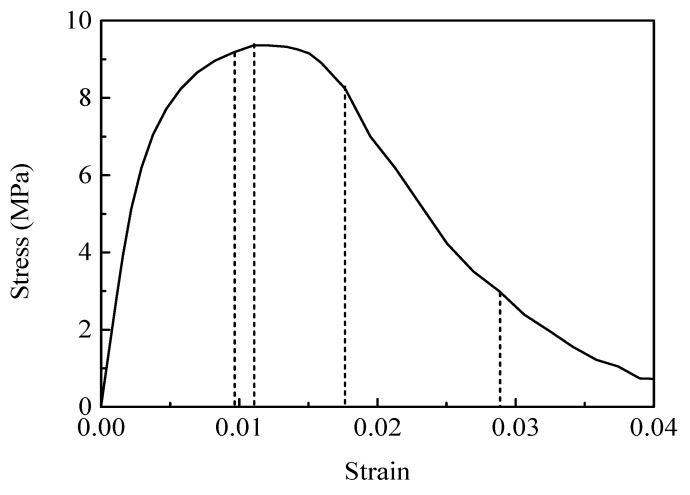
Compressive stress–strain curve of PBX 9501 at a strain rate of 10^−3^ s^−1^.

**Figure 20:**
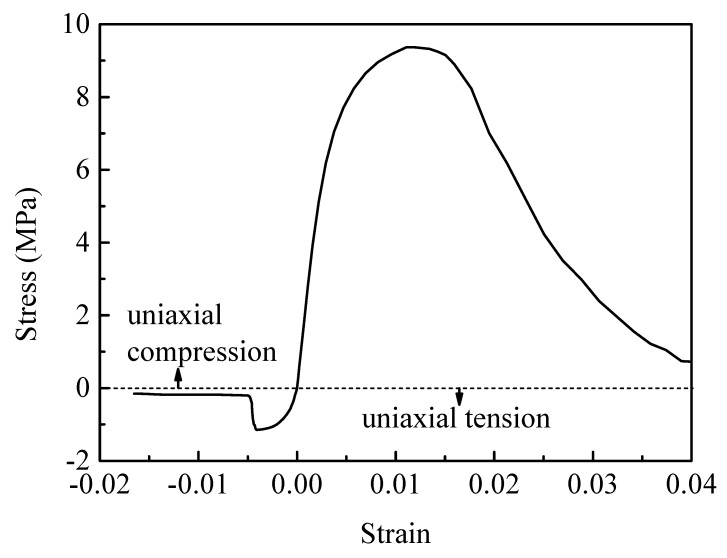
Stress–strain curve of PBX 9501 at a strain rate of 10^−3^ s^−1^.

The mechanical behavior of PBX 9501 under uniaxial compression is obviously different from that under uniaxial tension. Figure 19 plots the stress–strain response of PBX 9501 at a compressive strain rate of 10^−3^ s^−1^. The similar response features with those under uniaxial tension (such as initial rapid growth, peak stress, and softening behaviors) can also be observed. While some notable differences still exist, as shown in Figure 20, the softening rate of PBX 9501 under uniaxial compression is lower than that under uniaxial tension. The peak strength and failure strain are approximately 9.36 MPa and 0.0118, respectively, which are higher than the 1.16 MPa and 0.0041 under uniaxial tension. This reveals that the compressive properties of PBX 9501 are greater than its tensile properties.

**Figure 21:**
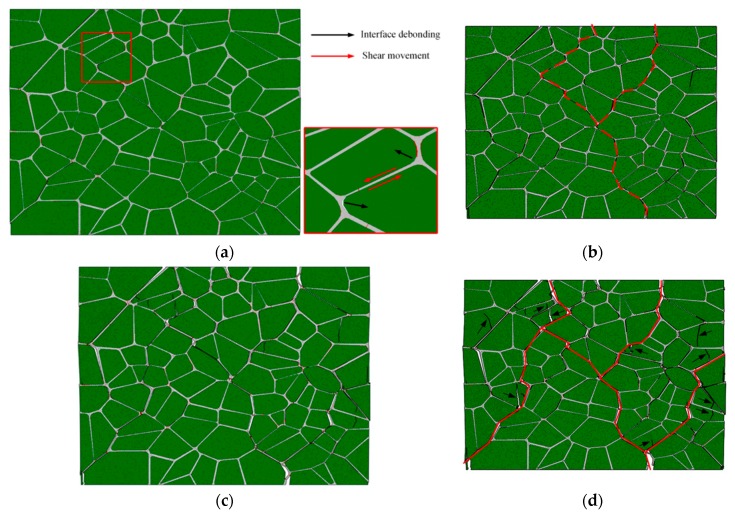
The failure evolution of PBX 9501 for different macroscopic strains, (**a**) 0.96%, (**b**) 1.2%, (**c**) 1.77%, and (**d**) 2.88%, under uniaxial compression at a strain rate of 10^−3^ s^−1^.

Figure 21 shows the failure evolution of PBX 9501 at a macroscopic strain of 0.96%, 1.2%, 1.77%, and 2.88%, for a 10^−^^3^ s^−^^1^ strain rate. At the early stage of loading, the strain increases from 0% to 0.96%, and the soft binder sustains primarily deformation. Debonding occurs along the relatively weak particle/binder interface due to its shearing slide, as shown in Figure 21a. Thus, the tangential stress of the interface first reaches its critical value under compression loading. With an increase in loading, the compression strength reaches its maximum, and more interface microcracks and transgranular fractures of the particle occur (see Figure 21b,c) due to the interface slide and complicated interactions between the particles. Indeed, this interaction affects the failure mechanism of PBX 9501. Next, at a macroscopic strain of 2.88%, the macroscopic failure paths labeled by the red line spread over the whole model, along with the growth and connection of numerous microcracks, so that relatively shear failure modes are exhibited. In Figure 21, we can see that interface debonding and particle fractures (marked by arrows) are the primary modes of failure under uniaxial compression. These results were also observed in an experiment by Gray et al. and Zhou et al. [40,41].

#### 4.2.2. Effect of Particle/Binder Interface Strength

**Figure 22:**
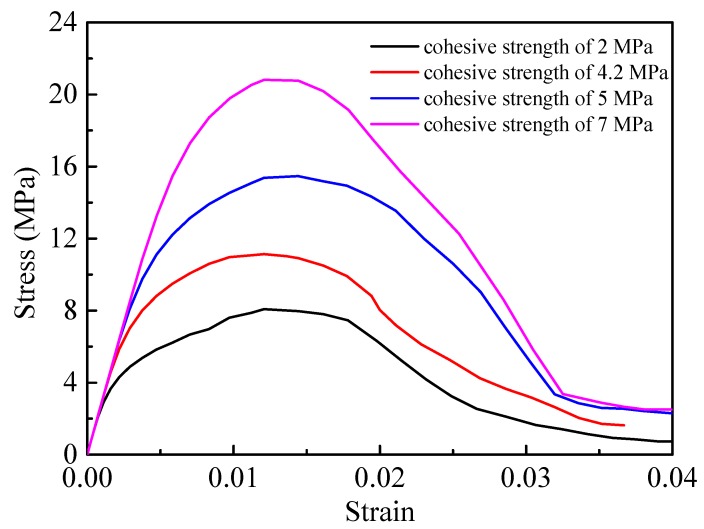
Stress–strain curves of the models with different interface strengths under uniaxial compression at a strain rate of 10^−^^2^ s^−1^.

The effect of the interface strength on the mechanical response of PBX 9501 at a compressive strain rate of 10^−^^2^ s^−1^ was also investigated. Four different tangential strengths of the particle/binder interface, including 2 MPa, 4.2 MPa, 5 MPa, and 7 MPa, were selected for the model in Figure 10b. Figure 22 plots the compressive stress–strain curves for a strain rate of 10^−^^2^ s^−1^ at different interface strengths. As the interface strength becomes stronger, the compressive strength becomes higher. Thus, the failure strain is relatively independent of the interface strength.

**Figure 23:**
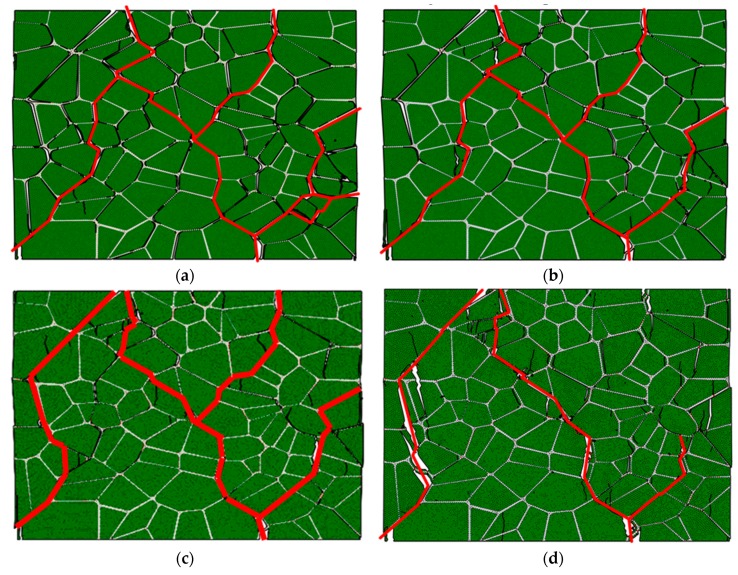
The failure path for different interface strengths, (**a**) 2 MPa, (**b**) 4.2 MPa, (**c**) 5 MPa, and (**d**) 7 MPa under compression loading.

Figure 23 presents the failure paths for different interface strengths under compression loading. As seen in Figure 23a–d, a large number of microcracks along the interface reveals that interface debonding is the primary failure mode for the weaker interface strength. With an increase of the interface strength, the particle fracture becomes more obvious, especially when the interface strength is 7 MPa. This suggests that a stronger interface strength prevents the relative motion of particle/binder interfaces, thereby enhancing the interactions between particles and increasing the possibility of a particle’s fracture. The simulation results agree with Wu’s predicted results [24].

#### 4.2.3. Effect of the Strain Rate

**Figure 24:**
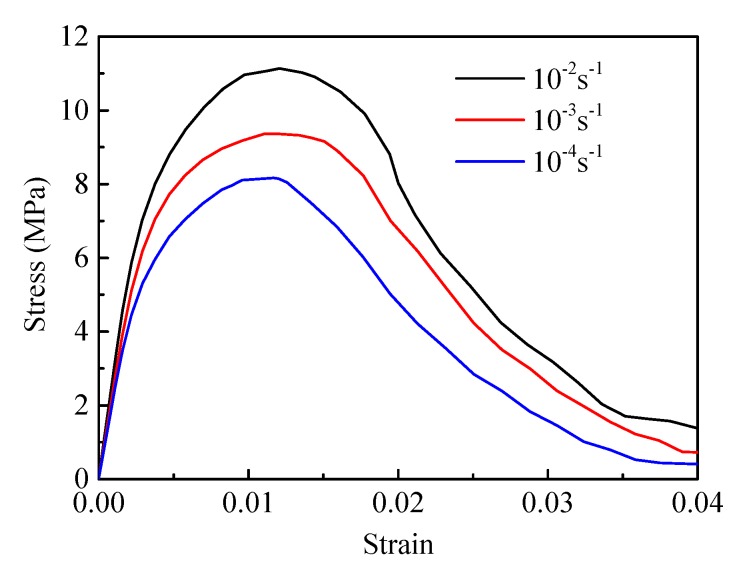
Compressive stress–strain curves of PBX 9501 at different strain rates.

The compressive properties of PBX show obvious strain rate dependence, which has also been reported by Gray et al. and Chen et al. [8,40]. We carried out a series of simulations under three different strain rates by controlling the displacement loading rate. Figure 24 shows the stress–strain curves for strain rates of 10^−4^ s^−1^, 10^−3^ s^−1^, and 10^−2^ s^−1^. As can be seen from the figure, the compressive strength increases as the strain rate increases, while the failure strain has almost no significant change compared with the compressive strength. Based on this phenomenon, Wiegand et al. proposed a constant critical strain failure criterion for PBXs [42].

**Figure 25:**
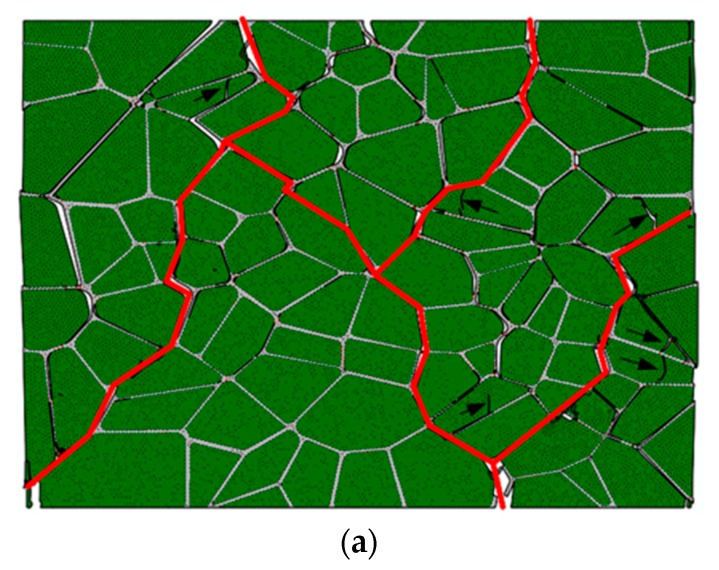

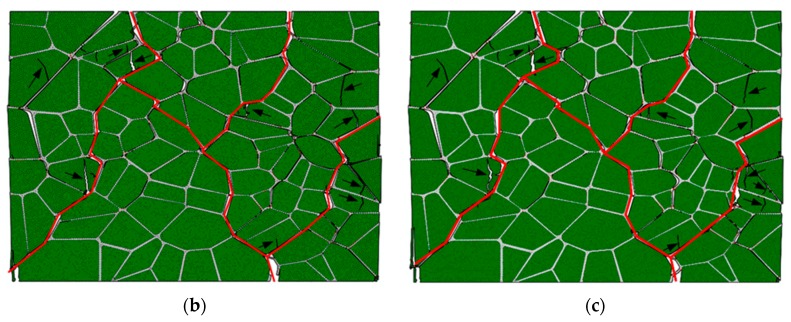
The failure path of PBX 9501 under compression loading at three strain rates: (**a**) 10^−^^4^ s^−^^1^, (**b**) 10^−^^3^ s^−^^1^, and (**c**) 10^−^^2^ s^−^^1^.

Figure 25 shows the failure path of PBX 9501 under compression loading at three strain rates. Clearly, the failure path is similar, as indicated by the red line, which also illustrates that the failure path is insensitive to strain rate. The higher the strain rate, the more serious the particle failure (marked by arrows) is. This may explain why the stiffening in the binder at a high strain rate strengthens particle interactions in compression. This seems to imply that a stronger binder can increase the possibility of the particle’s fracture.

## 5. Conclusions

We have presented a method that embeds cohesive elements throughout a microstructure model obtained by Voronoi tessellation, to study the response of PBX 9501 subjected to quasi-static loading. The calculated stress–strain curves agree well with the experimental results. PBX 9501 can be considered a brittle material with a low failure strain. The compressive strength of PBX 9501 is much higher than its tensile strength. The random particle size shape and interface debonding behavior of PBX 9501 produce local heterogeneous strain and failure distribution. Different failure mechanisms are observed. Interface debonding is the primary failure mode of the model under tensile loading. Under compressive loading, however, interface debonding and particle fracture play important roles.

We also used this model to study the effects of interface strength and strain rate on the mechanical properties and failure evolution of PBX 9501. The simulation results show that the higher strength of the particle/binder interface corresponds to greater tensile and compressive strength. The interface strength only affects the tensile failure strain but not the compressive failure strain. The compressive strength of PBX 9501 is rate dependent, while its failure strain is insensitive to the strain rate. The failure evolution path is relatively independent of the interface strength and strain rate. At a high interface strength or strain rate, a stronger interface force or binder can increase the probability of particle failure. These obtained results are important for a good understanding of the material’s failure mechanisms and also useful for designing and improving the properties of advanced PBX composite materials by establishing the relationship between their meso-structures and performance.

## Figures and Tables

**Table 1:** Relaxation and shear Prony terms for Estane 5703.

**Shear stress relaxation Prony terms (MPa)**	*G*_∞_	*G*_1_	*G*_2_	*G*_3_	*G*_4_	*G*_5_	*G*_6_	*G*_7_
	0	75.8	117.489	177.828	251.188	346.737	457.088	436.516
	*G*_8_	*G*_9_	*G*_10_	*G*_11_	*G*_12_	*G*_13_	*G*_14_	*G*_15_
	223.872	52.481	12.882	2.618	2.104	0.4753	0.2218	0.1622
	*G*_16_	*G*_17_	*G*_18_	*G*_19_	*G*_20_	*G*_21_	*G*_22_	
	0.0056	0.0891	0.0676	0.03802	0.01585	0.00741	0.00417	
**Relaxation time (s)**	*τ*_1_	*τ*_2_	*τ*_3_	*τ*_4_	*τ*_5_	*τ*_6_	*τ*_7_	*τ*_8_
	7.35 × 10^−16^	7.35 × 10^−15^	7.35 × 10^−14^	7.35 × 10^−13^	7.35 × 10^−12^	7.35 × 10^−11^	7.35 × 10^−10^	7.35 × 10^−9^
	*τ*_9_	*τ*_10_	*τ*_11_	*τ*_12_	*τ*_13_	*τ*_14_	*τ*_15_	*τ*_16_
	7.35× 10^−8^	7.35 × 10^−7^	7.35 × 10^−6^	7.35 × 10^−5^	7.35 × 10^−4^	7.35 × 10^−3^	7.35 × 10^−2^	7.35 × 10^−1^
	*τ*_17_	*τ*_18_	*τ*_19_	*τ*_20_	*τ*_22_	*τ*_23_		
	7.35	7.35 × 10^1^	7.35 × 10^2^	7.35 × 10^3^	7.35 × 10^4^	7.35 × 10^5^		

**Table 2:** Parameters for the three types of cohesive elements.

Cohesive Element Type		Normal		Tangential		$\delta_{n}^{f},\delta_{t}^{f}(\mathrm{mm})$
		$k_{n}\;(\mathrm{GPa}/\mu m)$	$T_{nc}\;(\mathrm{MPa})$	$k_{t}\;(\mathrm{GPa}/\mu m)$	$T_{tc}\;(\mathrm{MPa})$	
Particle		2	4.754	55	131	0.005
Binder		1	5	7.68	38.4	5
Particle–binder interface	10^−4^ s^−1^	1.55	1.15	3.3	2.46	0.084
	10^−3^ s^−1^	1.55	1.66	3.3	3.55	0.11
	10^−2^ s^−1^	1.55	4.2	3.3	8.98	0.17

**Table 3:** Calculated peak stress and failure strain.

Element Size (μm)	Tensile Loading/10^−3^ s^−1^		Compressive Loading/10^−2^ s^−1^	
	Peak Stress (MPa)	Failure Strain	Peak Stress (MPa)	Failure Strain
5	1.17	0.0041	11.17	0.0118
8	1.16	0.0041	11.13	0.012
10	1.11	0.0043	10.95	0.0124
error	0.85–5.13%	0–4.88%	0.36–1.97%	1.7–5.08%
